# Supplementary material for: The role of positively charge poly-L-lysine in the formation of high yield gold nanoplates on the surface for plasmonic sensing application
Source: PLoS One. 2021 Nov 8;16(11):e0259730. doi: 10.1371/journal.pone.0259730 (PMC8575294; doi:10.1371/journal.pone.0259730)
Supplement: S1 Fig — Scale (A): 10 μm and (B) 100 nm. (DOCX) [file pone.0259730.s001.docx]

**Supporting Document**

**S1 Fig. The FESEM image and yield percentage for the sample triple PLL layer. Scale (A): 10 µm and (B)100 nm**
