# Supplementary material for: The role of positively charge poly-L-lysine in the formation of high yield gold nanoplates on the surface for plasmonic sensing application
Source: PLoS One. 2021 Nov 8;16(11):e0259730. doi: 10.1371/journal.pone.0259730 (PMC8575294; doi:10.1371/journal.pone.0259730)
Supplement: Attachment — Submitted filename: Response to Reviewers.pdf [file pone.0259730.s002.pdf]

Manuscript PONE-D-21-28832

The Role of Positively Charge Poly-L-Lysine in the Formation of High Yield Gold Nanoplates on the Surface for Plasmonic Sensing Application
PLOS ONE

Response to reviewers' comments

We are very grateful for the reviews provided by the editor and each of the external reviewers of this manuscript. The comments are encouraging, and the reviewers appear to share our judgement that this study and its results are clinically important. Please see below, our detailed response to comments. All page numbers and line are referred to the manuscript file.

Academic Editor

No	Comments	Correction and Explanation
1	Please ensure that your manuscript meets PLOS ONE's style requirements, including those for file naming	The manuscript have been changed to follow PLOS ONE's style requirements, including those for file naming
2	We suggest you thoroughly copyedit your manuscript for language usage, spelling, and grammar. If you do not know anyone who can help you do this, you may wish to consider employing a professional scientific editing service. Upon resubmission, please provide the following:<br><br>The name of the colleague or the details of the professional service that edited your manuscript<br><br>A copy of your manuscript showing your changes by either highlighting them or using track changes (uploaded as a *supporting information* file)<br><br>A clean copy of the edited manuscript (uploaded as the new *manuscript* file)"	The manuscript have been proofread
3	We note that the grant information you provided in the 'Funding Information' and 'Financial Disclosure' sections do not match	The funding information as stated below;<br><br>The research is supported by Ministry of Higher Education (MOHE) Malaysia through Fundamental Research Grant Scheme (FRGS/1/2019/STG07/UTHM/02/1) (MM, FM) (https://mygrants.gov.my) and Hibah Penelitian GBU45 (No. B/515/IT9. C/PT.01.03/2021) (SN) from Institut Teknologi Sumatera. The authors would also like to thank Universiti Tun Hussein Onn Malaysia for partially sponsored this work.

		This statement has been added in cover letter for amendment.
4	4. Thank you for stating the following in the Acknowledgments Section of your manuscript:<br><br>"The research is funded by FRGS grant (FRGS/1/2019/STG07/UTHM/02/1) from the Ministry of Education (MoE) Malaysia and Hibah Penelitian GBU45 (No. B/515/IT9. C/PT.01.03/2021) from Institut Teknologi Sumatera. The authors also would like to thank to the Universiti Tun Hussein Onn Malaysia for partially sponsored this work. The experiment was conducted at Microelectronics & Nanotechnology - Shamsuddin Research Centre (MiNT-SRC), UTHM."<br><br>We note that you have provided funding information that is not currently declared in your Funding Statement. However, funding information should not appear in the Acknowledgments section or other areas of your manuscript. We will only publish funding information present in the Funding Statement section of the online submission form.<br>Please remove any funding-related text from the manuscript and let us know how you would like to update your Funding Statement. Currently, your Funding Statement reads as follows:<br><br>"The research is funded by FRGS grant (FRGS/1/2019/STG07/UTHM/02/1) (MM, FM) from the Ministry of Education (MoE) Malaysia (https://mygrants.gov.my) and Hibah Penelitian GBU45 (No. B/515/IT9. C/PT.01.03/2021) from Institut Teknologi Sumatera. The authors also would like to thank to the Universiti Tun Hussein Onn Malaysia for partially sponsored this work. The experiment was conducted at Microelectronics & Nanotechnology - Shamsuddin Research Centre (MiNT-SRC), UTHM"	The funding statement has been removed in acknowledgement section.

Reviewer 1

In this manuscript, the authors proposed an anisotropic structure, gold (Au) nanoplates was synthesized using a two steps wet chemical seed mediated growth method (SMGM) directly on the substrate surface. They claimed that the high yield of Au nanoplates exhibit dual plasmonic peaks response that are associated with transverse and longitudinal localized surface plasmon resonance (TSPR and LSPR). Furthermore, the proposed structure is novel, and the results are valuable and exciting to the readers. However, the necessary references and physical mechanism are absent in the manuscript to explain the related results. In summary, I recommend that the manuscript undergo a major revision to address my comments below before resubmission to this journal.

No	Comments	Correction and Explanation
1	Line 31-33, it is written that "The high yield of Au nanoplates … are associated with transverse and longitudinal localized surface plasmon resonance (tspr and lspr). The "(tspr and lspr)" should be used capital letters (TSPR and LSPR).	The tspr and lspr have been changed to capital letters (TSPR and LSPR).
2	Line 55-57, it is written that "The physical method requires high equipment costs and a lengthy preparation process such as lithography [7]". The suitable reference (Nanomaterials (2019),9(12),1691 and Results in Physics (2020) 17, 103116) are suggested to quote regarding this sentence.	The suggested references (Nanomaterials (2019),9(12),1691 and Results in Physics (2020) 17, 103116) have been cited in the manuscript.<br>Line 59-60:<br>The physical method requires high equipment costs and a lengthy preparation process such as lithography [7-8]
3	"Currently, our group have been working with AuNPs specifically in sensing application." The related articles from your group should be quoted with respect to this sentence.	Our works with AuNPs specifically in sensing application have been cited in this manuscript.<br>Line 78-79:<br>Currently, our group have been working with AuNPs specifically in sensing application [24-28].
4	Line 79-80, it is written that "The sensing properties is strongly depending on the size and shape in producing their plasmon band." The suitable references (J. Electromagn. Waves Appl. (2010) 24(8-9) 1005-1014 and Results in Physics (2019) 13, 102290) are suggested to be cited after this sentence.	References; Results in Physics (2019) 13, 102290) and Analyst, 140(2), 386-406 has been included as references and cited in the manuscript.<br><br>J. Electromagn. Waves Appl. (2010) 24(8-9) 1005-1014 is not very suitable because the article discuses more on silver and the publication date is more than 5 years.<br><br>Line 81-82:<br>The sensing properties is strongly depending on the size and shape in producing their plasmon band [29-30].
5	Line 114, it is written that "The chemicals used for the synthesis are same as published in [Procedia]". What is [Procedia]? Is it a reference? Please check this sentence.	This is technical error and the Procedia have been replaced by ref no.17<br>Line 113<br>17. Procedia engineering, 2017; 184, 637-642.

6	Line 124-126, it is written that" The samples were prepared for 6 different sets of PLL 0.01 % (w/v) concentration; 0 %, 1 %, 5, %, 10 % and labelled as PL0, PL1, PL5 and PL10." Only four different sets are presented in this sentence. Please check it. Besides, it suggests replacing "6" as "six".	The number is corrected to four different sets.<br>Line 123-124:<br>The samples were prepared for four different sets of PLL 0.01 % (w/v) concentration;
7	Line 175-176, it is written that" Table 1 listed all the intensity and angles for all samples." Please clarify in more detail that how to measure the intensity.	The reading of the intensity is measured by capturing the highest intensity (a.u) from the data. To prevent misunderstanding, the value of intensity has been removed from the graph because the results are arranged in order to make it clearly view, not starting from the original. The captured data is tabulated in Table 1. The sentence has been added to the paragraph.<br>Line 181-183:<br>The intensity is obtained by observing the highest peaks detected by XRD for each samples and the results have been arranged in sequence for clearer view.
8	Line 217-218, it is written that" The two peaks assigned as transverse SPR (tspr) for the first peaks and the second peak is longitudinal SPR (lspr). The tspr is free charges vibration in vertical direction of the AuNPs on the surface and lspr is vibration of free charges in the horizontal direction, i.e., parallel with substrate surface." I think the explanation of the mechanism is not enough. To help the readers to understand the nature of LSPR and TSPR, authors can refer to "J. Appl. Phys. (2016), 120(9), 093110" and "Plasmonics (2008), 3(4), 157-164" or quote them for simplicity.	The additional explanation has been added as follow;<br><br>Line 225-230:<br>The interaction between incident electromagnetic wave with nanogold surface has been explained by Chou Chau et al [44]. The peak resonant wavelengths are determined by elemental composition and aspect ratio using Drude model [45]. The transverse modes contribute higher intensity with the longitudinal modes dominate the field localization and the transverse modes contribute the field radiation on the surface of the anisotropic nanoparticles.<br>44. *Journal of Applied Physics* 120, no. 9 (2016): 093110.<br>45. Kreibig, Uwe, and Michael Vollmer. *Optical properties of metal clusters*. Vol. 25. Springer Science & Business Media, (2013), p. 100.<br><br>The suggested reference; Plasmonics (2008), 3(4), 157-164 is not very suitable to be cited because the article discuses more on silver and the publication date is more than 5 years.
9	Line 221-230, the authors deduce the surface positive-negative charge pairs corresponding to Fig. 6. The simulation results are suggested to explain this point. If the authors cannot perform the simulations, they can quote the related	The surface positive-negative charge pairs corresponding to Fig. 6 has been further discussed in the paragraph.<br><br>Line 235-245:

	articles for simplicity (e.g., J. Nanopart. Res. (2018) 20(7), 190 and Nanoscale Research Letters (2016), 11(1),41)	The direct binding of PLL with Au has not strong electrostatics interactions and it can be improved by assisting the PLL with citrate-capped gold nanoseeds. As a result, the strong electrostatic interactions occurred due to weak acidic condition from citrate [46]. The schematic of positive charge layer with variation concentration of PLL has been deducted in Fig 6. At 0 % concentration of PLL, no positive charged layer was formed. When the concentration increased to 1 %, the monolayer positive charged was formed on the substrate and when the concentration increased up to 5 %, the substrate surface was covered with homogeneous positive charged layer. However, when we increase the concentration to 10 %, the positive charges are increased. Hence, the PLLs tend to aggregate each other since the spacing is very narrow causing the non-homogenous positive layer. This situation resulting a phenomenon named Ostwald ripening [47] that the change of an inhomogeneous gold nanoplates on the surface structure over time.<br>46. Stobiecka, M. and Hepel, M., 2011. Double-shell gold nanoparticle-based DNA-carriers with poly-L-lysine binding surface. *Biomaterials*, *32*(12), pp.3312-3321.<br>47. Pattadar, D.K. and Zamborini, F.P., 2019. Effect of size, coverage, and dispersity on the potential-controlled Ostwald ripening of metal nanoparticles. *Langmuir*, *35*(50), pp.16416-16426.<br><br>The suggested references are not very suitable because;<br>i. J. Nanopart. Res. (2018) 20(7), 190 - focusing on Ag nanoparticles<br>ii. Nanoscale Research Letters (2016), 11(1),41) - Fe-Doped TiO_2 Nanoparticles<br>and not related with the PLL binding with Au capped citrate as discussed in this manuscript
10	The references used in the text should be improved. To be beneficial for the readers to know the other approaches of plasmonic sensors, the suggested articles need to be included in the suitable place of introduction section, i.e.,<br>a. Nanomaterials (2020), 10(3), 493, - Ag sensor<br>b. Results in Physics (2019) 15, 102567, - Ag nanorods<br>c. J. Nanopart. Res. (2020) 22(9), 297 – Ag squares,<br>d. Nanomaterials, 2020, 10(7), 1399- Ag nanorods<br>e. J. Phys. D: Appl. Phys. (2021) 54(11) 115301 – Ag nanorods	The references for anisotropic gold plasmonic sensor has been added in the manuscript as follow;<br><br>Line 334-336:<br>Additionally, the anisotropic Au nanoparticles has been intensively used for detection of targeted analyte that showing the potential of their sensing ability [51,52].<br>51. Pattanayak, S. and Jana, S.K., 2018. Controllable aqueous synthesis of near-IR-plasmonic anisotropic gold nanoparticles in the hydrazine concentration assisted: hydrazine-citrate hydrogen-bonded network at room temperature and application in highly sensitive SERS-based detection of Pb (II) species. *Inorganic and Nano-*

	f. Nanomaterials, 2020, 10(3), 493 – Ag nanohair<br>g. Results in Physics (2019) 15, 102567- Ag veins<br><br><br><br><br><br><br><br>Besides, please recheck the typos throughout the text.	*Metal Chemistry*, *48*(11), pp.535-540.<br>52. Sharifi, M., Hosseinali, S.H., Alizadeh, R.H., Hasan, A., Attar, F., Salihi, A., Shekha, M.S., Amen, K.M., Aziz, F.M., Saboury, A.A. and Akhtari, K., 2020. Plasmonic and chiroplasmonic nanobiosensors based on gold nanoparticles. *Talanta*, *212*, p.120782.<br><br>The suggested references are more on silver Ag nanoparticles.<br>a. Nanomaterials (2020), 10(3), 493, - Ag sensor<br>b. Results in Physics (2019) 15, 102567, - Ag nanorods<br>c. J. Nanopart. Res. (2020) 22(9), 297 – Ag squares,<br>d. Nanomaterials, 2020, 10(7), 1399- Ag nanorods<br>e. J. Phys. D: Appl. Phys. (2021) 54(11) 115301 – Ag nanorods<br>f. Nanomaterials, 2020, 10(3), 493 – Ag nanohair<br>g. Results in Physics (2019) 15, 102567- Ag veins<br><br>The typing errors have been corrected and this paper has been proofread.

Reviewer 2

This paper describes the design and it also provides some details of a boric acid sensor based on non-spherical gold nanoparticles (plates) attached to a solid surface.

No	Comments	Correction and Explanation
1	The sensitivity of such sensor to boric acid is low and has little practical importance.	The explanation has been included in text as follow;<br><br>Line 326-328:<br>Even though the LOD is low as compared to other complicated sensing techniques, the sensing response is fast and in order to enhance the sensitivity, the size and shapes control of gold nanoplates must be studied.
2	In contrast to what the authors are stating, the chemistry of nanoparticle stabilization on the surface coated with poly-lysine is more complex than simple electrostatic interaction. This had to be reviewed (this literature is available) and included in the discussion.	The electrostatic interaction between PLL and citrate-capped gold nanoseeds has been discussed with suitable references.<br><br>Line 235-245:<br>The direct binding of PLL with Au has not strong electrostatics interactions and it can be improved by assisting the PLL with citrate-capped gold nanoseeds. As a result, the strong electrostatic interactions occurred due to weak acidic condition from citrate [46]. The schematic of positive charge layer with variation concentration of PLL has been

		deducted in Fig 6. At 0 % concentration of PLL, no positive charged layer was formed. When the concentration increased to 1 %, the monolayer positive charged was formed on the substrate and when the concentration increased up to 5 %, the substrate surface was covered with homogeneous positive charged layer. However, when we increase the concentration to 10 %, the positive charges are increased. Hence, the PLLs tend to aggregate each other since the spacing is very narrow causing the non-homogenous positive layer. This situation resulting a phenomenon named Ostwald ripening [47] that the change of an inhomogeneous gold nanoplates on the surface structure over time.<br>46. Stobiecka, M. and Hepel, M., 2011. Double-shell gold nanoparticle-based DNA-carriers with poly-L-lysine binding surface. *Biomaterials*, *32*(12), pp.3312-3321.<br>47. Pattadar, D.K. and Zamborini, F.P., 2019. Effect of size, coverage, and dispersity on the potential-controlled Ostwald ripening of metal nanoparticles. *Langmuir*, *35*(50), pp.16416-16426
3	The reproducibility of measurements is inadequately described in this manuscript	The reproducibility test has been cited in the text by referring our previous work [43].<br><br>Line 328-330:<br><br>Instead of that, based on our previous works, the sensor shows good repeatability response by showing fast response and recovery for at least for five cycles [43].<br><br>43. Sensors, 17(5), p.947.
4	The paper is poorly written, with multiple grammatical errors.	The typing errors have been corrected and this paper has been proofread.
